# Delirium epidemiology in critical care (DECCA): an international study

**DOI:** 10.1186/cc9333

**Published:** 2010-11-23

**Authors:** Jorge I Salluh, Márcio Soares, José M Teles, Daniel Ceraso, Nestor Raimondi, Victor S Nava, Patrícia Blasquez, Sebastian Ugarte, Carlos Ibanez-Guzman, José V Centeno, Manuel Laca, Gustavo Grecco, Edgar Jimenez, Susana Árias-Rivera, Carmelo Duenas, Marcelo G Rocha

**Affiliations:** 1 Intensive Care Unit and Postgraduate Program, Instituto Nacional de Câncer, 10° Andar; Praça Cruz Vermelha, 23; Rio de Janeiro-RJ; CEP: 20230-130, Brazil; 2 Intensive Care Unit, Hospital da Bahia, Av. Prof. Magalhaes Neto, 1541, Pituba. Cep:41830-030, Salvador, Bahia, Brazil; 3 Intensive Care Unit, Hospital Juan A. Fernandez, Cervino 3356, Buenos Aires (ZIP-1425), Argentina; 4 Postgraduate Program Critical Care, Morones Prieto 3000 Doctores, 64710 Monterrey, Nuevo León, Mexico; 5 Intensive Care Unit Hospital del Salvador y Clínica INDISA, Avenida Santa María 1810, Providencia, Zip 7500000, Santiago, Chile; 6 Intensive Care Unit, Unidad de Terapia Intensiva Hospital Obrero N 1 Av Brasil s/n CP 8908, La Paz, Bolivia; 7 Intensive Care Unit, Hospital Luis Vernaza, Ext. 2005 Julián Coronel y Loja, 2560300, Guayaquil, Ecuador; 8 Intensive Care Unit, Hospital Naval, Avenida Santos Chocano s/n, CP 210001, Lima, Peru; 9 Intensive Care Unit, Sanatorio Americano, 2466 Isabelino Bosch, CP 11600, Montevideo, Uruguay; 10 Intensive Care Unit, Orlando Regional Medical Center, 86 W. Underwood, MP 80, Orlando, FL 32806, USA; 11 Intensive Care Unit, Hospital Universitario de Getafe, Carretera de Toledo Km 12,500, Getafe, 28905, Madrid, Spain; 12 Intensive Care Unit and Postgraduate Program, Universidad de Cartagena, Nuevo Hospital Bocagrande, Calle 5 kra 6, Cartagena, 57, Colombia; 13 Intensive Care Unit, Pavilhão Pereira Filho, Santa Casa de Misericórdia de Porto Alegre, Rua Annes Dias 285 CEP-90020, Porto Alegre, Brazil

## Abstract

**Introduction:**

Delirium is a frequent source of morbidity in intensive care units (ICUs). Most data on its epidemiology is from single-center studies. Our aim was to conduct a multicenter study to evaluate the epidemiology of delirium in the ICU.

**Methods:**

A 1-day point-prevalence study was undertaken in 104 ICUs from 11 countries in South and North America and Spain.

**Results:**

In total, 975 patients were screened, and 497 fulfilled inclusion criteria and were enrolled (median age, 62 years; 52.5% men; 16.7% and 19.9% for ICU and hospital mortality); 64% were admitted to the ICU because of medical causes, and sepsis was the main diagnosis (*n *= 76; 15.3%). In total, 265 patients were sedated with the Richmond agitation and sedation scale (RASS) deeper than -3, and only 232 (46.6%) patients could be evaluated with the confusion-assessment method for the ICU. The prevalence of delirium was 32.3%. Compared with patients without delirium, those with the diagnosis of delirium had a greater severity of illness at admission, demonstrated by higher sequential organ-failure assessment (SOFA (*P *= 0.004)) and simplified acute physiology score 3 (SAPS3) scores (*P *< 0.0001). Delirium was associated with increased ICU (20% versus 5.7%; *P *= 0.002) and hospital mortality (24 versus 8.3%; *P *= 0.0017), and longer ICU (*P *< 0.0001) and hospital length of stay (LOS) (22 (11 to 40) versus 7 (4 to 18) days; *P *< 0.0001). Previous use of midazolam (*P *= 0.009) was more frequent in patients with delirium. On multivariate analysis, delirium was independently associated with increased ICU mortality (OR = 3.14 (1.26 to 7.86); CI, 95%) and hospital mortality (OR = 2.5 (1.1 to 5.7); CI, 95%).

**Conclusions:**

In this 1-day international study, delirium was frequent and associated with increased mortality and ICU LOS. The main modifiable risk factors associated with the diagnosis of delirium were the use of invasive devices and sedatives (midazolam).

## Introduction

Delirium is a common cause of acute brain dysfunction in patients admitted to the intensive care unit (ICU) [1,2]. To date, several studies have demonstrated that delirium is associated with increased mortality as well as increased hospital length of stay (LOS) and costs [2-4]. In addition, when high-risk populations are considered, such as the elderly and mechanically ventilated, delirium may occur in up to 80% of ICU patients [5]. The impact of delirium on relevant clinical outcomes is not restricted to the hospital setting, as delirium is also an independent predictor of 6-month mortality and long-term cognitive impairment [5,6]. However, most epidemiologic data derive from studies performed in one or a few centers in tertiary hospitals and academic centers where delirium awareness and adherence to best practice is probably increased [7]. Recent surveys involving large numbers of ICU healthcare professionals have demonstrated that despite the increasing knowledge of the pathophysiology, risk factors, and outcomes associated with delirium, it is still underdiagnosed, and modifiable risk factors related to its occurrence are frequently neglected [8,9]. However, these surveys were questionnaires that evaluated the perceptions and not the current practice of these professionals [8,9]. Therefore, it is important to describe and understand delirium epidemiology in a wide array of ICUs with different practice patterns. The availability of epidemiologic data from a large number of ICUs may help to design future observational and interventional studies. The aim of the present study was to evaluate the epidemiology of delirium in a large number of ICUs in South and North America and Spain.

## Materials and methods

### Design and setting

This 1-day observational study was performed on November 27, 2009, at 08:00 AM, local time, in 104 ICUs in Argentina, Bolivia, Brazil, Chile, Colombia, Ecuador, Mexico, Peru, Spain, the United States of America, and Uruguay. Pediatric ICUs, postoperative recovery areas, and units providing exclusive coronary care were not included. The institutional review boards approved the study design and waived the need for informed consent. The current study did not interfere with patient-management decisions.

### Selection of participants, data collection, and definitions

ICUs were recruited by using the mailing database from the study coordinator and the Federacion Panamericana e Iberica de sociedades de Medicina Critica y Terapia Intensiva (FPIMCTI). Each investigator and research coordinator was provided access to a website where a comprehensive manual describing data-collection requirements and variable definitions was available. A training manual for the Richmond Agitation and Sedation Scale (RASS) and Confusion Assessment Method for the ICU (CAM-ICU) in Portuguese, Spanish, and English, as well as videos demonstrating the application of the CAM-ICU, were available online for the investigators. A central office was accessible through telephone and email contact to answer questions regarding data collection on the study day and throughout the follow-up period. All data entry was performed online in a web-based electronic case report form (e-CRF). Data were checked by study coordinators to identify omissions, and inconsistent data were corrected whenever possible. ICU and hospital demographic information collected included the number of ICU beds, number of patients in the ICU at the moment of study, and number of patients meeting inclusion criteria. Patients were excluded from the study if they had a Glasgow coma scale < 14 from a primary neurologic diagnosis at ICU admission or before the study day on the same hospital admission or both. Legal blindness and deafness and the inability to speak the language of the country where the ICU was located and moribund patients (expected to die in less than 24 hours) were also exclusion criteria. All patients 18 years or older, with more than 24 hours of ICU stay were included regardless of the sedation status. The following information was collected in each patient meeting inclusion criteria on the day of the study: Gender, date of ICU and hospital admission, SAPS3 [10] and SOFA scores [11] at ICU admission, diagnosis, description of previous and current use of sedatives, and the use of antipsychotic agents during the ICU stay. The category of admission (surgical elective versus emergency versus medical) was noted. Sepsis was stratified according to the American College of Chest Physicians/Society of Critical Care Medicine Consensus Conference criteria [12], and acute lung injury (ALI) and acute respiratory distress syndrome (ARDS) were defined according to the American-European Consensus Conference criteria [13]. The presence of invasive procedures/monitoring and organ support was recorded. Level of arousal was measured by using the RASS score [14], which rates a patient's level of agitation/sedation on a 10-point scale ranging from -5 (unarousable, not responsive to voice or physical stimulation) to +4 (combative). Delirium was diagnosed with the CAM-ICU [2]. The CAM-ICU was developed for use in critically ill, intubated patients, and details can be found at the icudelirium website. The CAM-ICU is a validated delirium-detection tool with high sensitivity and specificity and high interrater reliability [1,2,5,15]. The CAM-ICU assesses four features of delirium: (1) acute onset or fluctuating course, (2) inattention, (3) disorganized thinking, and (4) altered level of consciousness. To be considered CAM-ICU positive, the subject must display features 1 and 2, and either 3 or 4. Vital status (alive/dead) at ICU discharge and study day 30 was registered.

### Data presentation and statistical analysis

Standard descriptive statistics were used. Continuous variables were reported as median (25% to 75% interquartile range (IQR)). Univariate analysis was used to identify factors associated with hospital mortality. Two-tailed *P *values < 0.05 were considered statistically significant. Univariate and multivariate logistic regression were used to identify factors associated with hospital mortality. Variables yielding *P *values < 0.2 by univariate analysis were entered into a forward multivariate logistic regression analysis. Multivariate analysis results were summarized by estimating odds ratios (ORs) and respective 95% confidence intervals (CIs). Possible interactions were tested. The area under the receiver-operating characteristic curve was used to assess the models' discrimination. The SPSS 13.0 software package (Chicago, IL) and Prism 3.0 (Graphpad, La Jolla, CA) were used for statistical analysis.

## Results

### Characteristics of the study population

**Figure 1:**
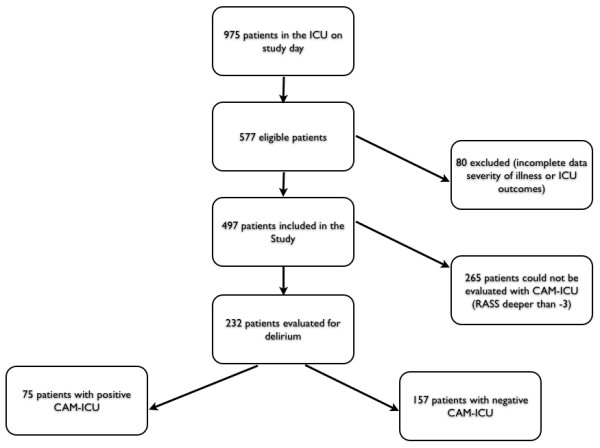
**Study flow chart**.

After the initial screening of 975, 497 patients that fulfilled entry criteria were enrolled in the study (Figure 1). Each institution of the DECCA database with its respective contributing proportion of patients is provided in Additional file 1. The main characteristics of the study population are depicted in Table 1. Overall, ICU and hospital mortality were 16.7% and 19.9%, respectively. Sixty-four percent were admitted to the ICU because of a medical condition, whereas elective and emergency surgery represented 21.5% and 14.1% of cases, respectively. At ICU admission, sepsis was the most frequent diagnosis (*n *= 76; 15.3%). Mechanical ventilation and vasopressors were used in 38.4% and 20.7% of the patients, respectively. Regarding chronic health status, 133 (26.7%) patients had a previous medical condition and required assistance.

**Table 1:** Demographic and clinical variables of patients according to delirium status

Variables	All patients (*n *= 497)	Delirium status^a^		*P *value
			
		Delirium (*n *= 75)	No delirium (n = 157)	
Age (years)	62 (47-74)	64 (50-77)	61 (46-74)	0. 2
Male gender, *n *(%)	261 (52.5%)	41 (54.6%)	79 (50.3%)	0.57
SAPS3 score (points)	49 (40-61)	57 (48-64)	46 (34-56)	< 0.0001
Charlson comorbidity index (points)	1 (0-3)	1 (0-3)	1 (0-3)	0.89
SOFA score (points)	4 (1-6)	4 (3-7)	3 (1-5)	0.004
Invasive mechanical ventilation, *n *(%)	191 (38.4%)	42 (56%)	36 (23%)	< 0.0001
Use of vasopressors, *n *(%)	103 (20.7%)	22 (29.3%)	21 (13.4%)	0.007
Renal replacement therapy, *n *(%)	52 (10.4%)	9 (12%)	17 (10.8%)	0.82
Main reasons for ICU admission				
Sepsis, *n *(%)	76 (15.3%)	19 (25.3%)	17 (10.8%)	0.006
Cardiovascular, *n *(%)	75 (15.3%)	10 (13.3%)	30 (18.6%)	0.35
Respiratory failure, *n *(%)	70 (11.7%)	9 (12%)	24 (15.3%)	0.55
Neurologic, *n *(%)	24 (4.8%)	12 (9.1%)	5 (3.1%)	0.004
Invasive devices				
Central venous catheter	317 (63.8%)	64 (85.3%)	85 (54.1%)	< 0.0001
Arterial catheter	158 (31.8%)	29 (38.6%)	32 (20.4%)	0.004
Urinary catheter	324 (65.1%)	62 (82.6%)	89 (56.7%)	0.0001
ICU LOS (days)	10 (4-24)	22 (11-40)	7 (4-18)	< 0.0001
ICU mortality, *n *(%)	83 (16.7%)	15 (20%)	9 (5.7%)	0.002
Hospital mortality, *n *(%)^b^	88 (19.9%)	18 (24%)	13 (8.3)	0.0017

The *P *values are for comparisons among patients with and without the diagnosis of delirium. ^a^Only those evaluated for delirium were considered. ^b^Only those with death or discharge at day 30 were considered (*n *= 711). SAPS3, Simplified Acute Physiology Score 3; SOFA, Sequential Organ Failure Assessment; ICU, intensive care unit; LOS, length of stay. Results are expressed as median (25% to 75% interquartile range) and number (%).

Among eligible patients, on the study day, 140 (20.8%) patients were receiving continuous infusion or regular administration of sedatives, and in 57 (40.7%) of the patients, interruption of sedation was performed as part of routine ICU care in these units. Considering only those using sedatives on the study day, the level of arousal was RASS > 1 in 10% (*n *= 14), RASS -1 to 1 in 35% (*n *= 49), and RASS ≤ 1 in 55% (*n *= 77). For these patients, sedation was considered by the assisting physician to be within the previously established target in 106 (75.7%) patients.

### Diagnosis of delirium: associated characteristics and outcomes

After excluding patients deeply sedated and unarousable with RASS deeper than -3, delirium was evaluated with the CAM-ICU in 232 patients (46.7% of the entire eligible patient population). Overall, delirium was diagnosed with the CAM-ICU in 75 (32.2%) of the included arousable patients. Detailed comparisons between patients with and without a diagnosis of delirium are depicted in Table 1. Patients with delirium were more severely ill, as reflected by higher SAPS3 and SOFA scores (*P *< 0.0001 and *P *= 0.004, respectively). In addition, patients with delirium had more frequent use of invasive mechanical ventilation, vasopressors as well as invasive devices, such as central venous and arterial catheters (Table 1). Additionally, patients with delirium used haloperidol more frequently (21.3% versus 3.8%; *P *< 0.0001) as compared with those without delirium. The overall use of atypical antipsychotics was low and similar in the two groups (5.3% versus 4.4%; *P *= 0.75). Regarding the use of sedatives during the ICU stay, only the use of midazolam was associated with the diagnosis of delirium (42.6% in patients with delirium versus 24.8% in those without the diagnosis of delirium; *P *= 0.009). Additional data on the use of sedatives is provided in Table 2.

**Table 2:** Use of sedatives in patients with and without a diagnostic of delirium

	Delirium (*n *= 75)	No delirium (*n *= 157)	*P *value
Midazolam	32 (42.6%)	39 (24.8%)	0.009
Other benzodiazepines	11 (14.68%)	20 (12.7%)	0.68
Fentanyl	26 (34.6%)	34 (21.6%)	0.15
Morphine	12 (16%)	21 (13.4%)	0.41
Propofol	12 (16%)	11 (7%)	0.058
Dexmedetomidine	12 (16%)	13 (8.3%)	0.11

Results are expressed as number and percentage. Only those evaluated by the CAM-ICU were included in the analysis.

Variables selected in the univariate analysis were entered into the multivariate analysis. As expected, potential collinearity between the SOFA and SAPS3 scores (Pearson's correlation coefficient, *r *= 0.43) was observed. Therefore, two models were fitted containing either the SAPS3 or the SOFA score. In addition to the SAPS3 and SOFA scores, delirium was selected in the final models and associated with ICU mortality (Table 3). On multivariate analysis, delirium was independently associated with increased ICU mortality (OR = 3.14 (1.26 to 7.86); CI, 95%) and hospital mortality (OR = 2.5 (1.1 to 5.7); CI, 95%).

**Table 3:** Multivariate analyses of factors associated with increased ICU mortality

Variables	Coefficient	Odds ratio (95% CI)	*P *value
*Model containing the SAPS3 score*			
Delirium	1.147	3.15 (1.26-7.86)	0.014
SAPS3 Score (points)	0.03	1.03 (0.99-1.06)	0.06
Constant	-4.309		
			
*Model containing the SOFA Score*			
SOFA Score (points)	0.14	1.14 (1.01-1.29)	0.023
Delirium	1.21	3.36 (1.36-8.29)	0.008

Constant	-3.384		

Model containing the SAPS3 Score: Area under receiver operating characteristic curve = 0.73 (95% CI, 0.67 to 0.79). Model containing the SOFA Score: Area under receiver operating characteristic curve = 0.75 (95% CI, 0.69 to 0.80). SAPS3, Simplified Acute Physiology Score 3; SOFA, Sequential Organ Failure Score; CI, confidence interval.

When patients with RASS deeper than -3 were analyzed, we observed that they had increased ICU mortality (*P *< 0.0001) and severity of illness (SAPS3, 49 (40 to 61] versus 46 (34 to 56); *P *= 0.01) but a similar age (62 (46 to 74) versus 61 (46 to 74); *P *= 0.8) as compared with patients without a diagnosis of delirium. When compared with those that were arousable and presented a diagnosis of delirium, deeply sedated patients had similar ICU mortality (*P *= 0.87) but a lower severity of illness (SAPS3, 49 (40 to 61) versus 57 (48 to 64); *P *= 0.0005) and a comparable age (62 (46 to 74] versus 64 (50 to 77); *P *= 0.28).

## Discussion

In this multicenter international study, we observed that, through a single standardized evaluation, delirium was diagnosed in 32% of the patients. Moreover, our data show that delirium was also associated with longer duration of hospitalization and was an independent predictor of ICU and hospital mortality. Considering the increasing costs associated with the ICU and hospital stay and the fact that delirium is often unrecognized [8,9,16], our findings have an increasing relevance. Additionally, mounting evidence suggests that delirium is associated with the risk of self-extubation, removal of catheters, and failed extubation, adverse events that are associated with worse outcomes [17]. Therefore, data from the present study showing its increased prevalence in academic and nonacademic centers, in private and public hospitals, as well as in different countries provide additional support to the recommendation for the use of a validated delirium-screening tool such as the CAM-ICU as a routine in the ICU [18,19].

The 32% incidence of delirium in the present study is comparable to that in previous reports from mixed ICU populations [4] but is lower than the incidence of around 80% observed in studies involving exclusively mechanically ventilated patients [5]. Such a significant difference may be ascribed to patients' characteristics (for example, case mix, disease severity, age), the tool used for delirium assessment, and sedation practices. Another aspect that could have influenced the present prevalence is related to the fact that patients in a coma or deeply sedated or both were not considered in the present study as they could not be evaluated with the CAM-ICU. Although coma and delirium are different clinical conditions, both can be classified as acute brain dysfunction [20]. Certainly, patients with delirium are prone to receive sedatives, especially when the hyperactive form is present; this could have led to a higher frequency of coma and oversedation but also to underestimation of the delirium rates in the present study.

Our findings have significant clinical and research implications. First, they confirm the previous findings from single-center studies showing that among medical/surgical ICU patients, delirium is associated with adverse outcomes, including prolonged ICU hospital stay, and is an independent predictor of increased short-term mortality [2,5,21]. Among factors associated with delirium in our study, invasive devices and the use of midazolam are to be considered potentially modifiable risk factors. Among sedatives, only midazolam reached statistical significance; however a trend was observed with propofol (*P *= 0.058) another γ-aminobutyric acid (GABA)-agonist sedative. The lack of association observed with other benzodiazepines may be explained by a type II error, as the study was probably underpowered to detect this association. Therefore, we consider that routine delirium assessment, judicious use of sedatives, and early removal of invasive devices (that is, catheters, drains, tubes) to be incorporated into the plan of care of critically ill adults. These and other strategies intended to decrease the frequency and severity of delirium have been successfully tested in non-ICU hospitalized high-risk patients (that is, restraint reduction, early device removal, frequent mobilization, hearing and visual aids, and efforts to improve patient communication through assistive strategies) [22] and should be implemented in the critical care setting.

Finally, different patterns of practice may play an important role in critical care outcomes [23]. Currently, a paucity of data exists regarding global prevalence and practice regarding delirium. In most published studies evaluating delirium, the enrolled patients are predominantly from North America and Europe, even though delirium in the ICU is a global challenge. In this regard, data from multicenter studies in different regions of the world are important to provide additional information and to allow better design of future clinical trials.

Our study has some shortcomings that must be addressed. First, it is a 1-day point-prevalence study, and potential seasonal selection bias cannot be ruled out. Nonetheless, enrolling a large number of ICUs usually diminishes this aspect. In addition, follow-up was restricted to 30 days; therefore, we were not able to address the impact of delirium on long-term morbidity and mortality of our population of critically ill patients. Even so, the present study provides solid data from a large number of ICUs in 11 countries demonstrating that delirium is not only prevalent but also independently associated with increased ICU LOS, mortality, and hospital mortality.

In a point-prevalence study, one must deem possible that other factors may affect patients' outcomes. One possible factor might be related to significant practice variation in delirium treatment [8,9,24]. Delirium is treated in various ways (that is, physical restraint, sedatives, antipsychotics), and such diverse approaches may have effects on the clinical outcomes evaluated in our study. Furthermore, in the present study, delirium was considered a dichotomous variable, a yes/no event. Thus, it is reasonable to consider that our results could have varied if delirium severity and duration were measured [5,25-27]. Regarding the factors associated with delirium in our study, the current design does not allow us to establish a true "cause/effect" relation between delirium and the selected outcomes. However, our multicenter study involving numerous ICUs does provide evidence of the negative effect of delirium on major clinical outcomes in mixed critically ill patients.

## Conclusions

This 1-day point-prevalence international study confirms previous findings from single-center studies showing that delirium occurs frequently and is independently associated with adverse outcomes in general ICU patients. Among clinical characteristics associated with the diagnosis of delirium, the use of invasive devices and midazolam were identified and may be considered potentially modifiable risk factors. The study provides a "real world" picture of delirium in general ICU patients in many different countries, and the data should prove useful in the design of trials of pharmacologic and nonpharmacologic interventions for delirium.

## Key messages

• The application of a single standardized evaluation may diagnose delirium in 32% of general ICU patients.

• The diagnosis of delirium is associated with worse outcomes including longer ICU and hospital length of stay and is independently associated with short-term mortality.

• The use of invasive devices and sedatives (midazolam) is associated with the diagnosis of delirium. These should be considered modifiable risk factors in the ICU, prompting the inclusion of a systematic evaluation for early device removal and judicious sedation in patients' plan of care.

## Abbreviations

ALI: acute lung injury; ARDS: acute respiratory distress syndrome; CAM-ICU: confusion-assessment method for the ICU; CI: confidence interval; ICU: intensive care unit; IQR: interquartile range; LOS: length of hospital stay; MV: mechanical ventilation; OR: odds ratio; RASS: Richmond agitation and sedation scale; SAPS3: Simplified Acute Physiology Score 3.

## Competing interests

The study was funded by the Federacion Panamericana e Iberica de sociedades de Medicina Critica y Terapia Intensiva (FPIMCTI). JIFS, JMT, and MGR have received honoraria and unrestricted research grants from Hospira, Inc. All other authors report that they have no competing interests.

## Authors' contributions

JIFS, MS, and MGR contributed to the study conception and design, carried out and participated in data analysis, and drafted the manuscript. All authors worked on patient inclusion and helped to revise the manuscript. All authors read and approved the final manuscript.

## Supplementary Material

Additional file 1 **A description of each institution of the DECCA database with its respective contributing proportion of patients**. Click here for file
