# Supplementary material for: Delirium epidemiology in critical care (DECCA): an international study
Source: Crit Care. 2010 Nov 23;14(6):R210. doi: 10.1186/cc9333 (PMC3220001; doi:10.1186/cc9333)
Supplement: Additional file 1 — A description of each institution of the DECCA database with its respective contributing proportion of patients. [file cc9333-S1.DOC]

Additional file 1. **Description of each institution of the DECCA database with its respective contributing proportion of patients.**

**Country Representation (%)**

Argentina 73 (7.48%)

Bolivia 6 (0.06%)

Brazil 435 (44.6%)

Chile 54 (5.5%)

Colombia 187 (19.18%)

Ecuador 41 (4.2%)

Mexico 95 (9.74%)

Peru 5 (0.05%)

Spain 28 (2.87%)

Uruguay 20 (2.05%)

USA 31 (3.18%)

Total 975 patients

***Investigators and affiliations*:**

**Argentina**: Dr. Alberto Legarto, Hospital Italiano de La Plata, La Plata, Argentina; Dr. Carlos Apezteguia, Hospital Posadas, Buenos Aires, Argentina; Dr. Cayetano Guillermo Galletti, Sanatório Allende, Cordoba, Argentina; Dr. Cayetano Guillermo Galletti, Sanatório Allende, Cordoba, Argentina; Dr. Cristina Orlandi, Clínica General Roca, Rio Negro, Argentina; Dr. Eloy Garcia, Hospital Regional de Comodoro Rivadavia, Comodoro, Argentina; Dr. Enrique Romero, Hospital Privado de Cordoba, Cordoba, Argentina; Dr. Fernando Pálizas, Sanatorio Sta. Isabel e Clínica Bazterrica, Buenos Aires, Argentina, Dr. Fernando Rios, Sant Las Lomas San Isidro, San Isidro, Argentina; Dr. Guillermo Chiappero, Hospital Universitario Austral, Pilar, Argentina; Dr. Jose Luis Do Pico, Hospital Municipal de Necochea, Necochea, Argentina; Dr. Jorge Arroyo, Hospital Central,Mendonza, Argentina; Dr. Jorge Neira, Sanatorio de La Trinidad, Buenos Aires, Argentina; Dr. Marcelo Prochasko, Hospital de Alta Complejidad de Formosa, Formosa, Argentina; Dr. Néstor Raimondi, Hospital Juan A. Fernandez, Buenos Aires, Argentina; Dr. Octavio Alejandro Guanca, Hospital Dr. Joaquín Castellanos, Salta, Argentina; Dr. Pascual Valdez, Hospital Vélez Sarfield, Buenos Aires, Argentina; Dr. Luciana Vanesa León Cejas, Hospital Pablo Soria, San Salvador de Jujuy, Argentina; Dr.Hernán Nuñez, Sanatorio San Lucas, San Isidro, Argentina.

**Bolivia:** Dr. Carlos Ibáñez Guzmán, Hospital OBRERO Nº 1, La Paz, Bolívia.

**Brazil:**Dr Alberto José de Barros Neto, Hospital Alfa e Real Hospital Português de Beneficência em Pernambuco, Recife, Brasil; Dr. Amadeu Martinez, Hospital Espanhol, Salvador, Brasil; Dr. André Gustavo, Hospital Quinta D'or, Rio de Janeiro, Brasil; Dr. André Miguel Japiassu, Instituto de Pesquisa Clínica Evandro Chagas, Rio de Janeiro, Brasil; Dr. André Torelly, Santa Casa de Porto Alegre - Hospital Santa Rita, Porto Alegre, Brasil; Dr Antonio Eiras Falcão, UTI-UNICAMP, Campinas, Brasil, Dr. Arthur Vianna, Clínica São Vicente, Rio de Janeiro, Brasil; Dr. Bruno da Silva Ferreira, Hospital Pasteur, Rio de Janeiro, Brasil; Dr. Carlos Eduardo Nassif Moreira, Hospital Nove de Julho, São Paulo, Brasil; Dra. Carmem Barbas, Hospital Israelita Albert Einstein e HC - Universidade de São Paulo, São Paulo, Brasil; Dra.Cassia Righy, Hospital Copa D'or, Rio de Janeiro, Brasil; Dr. Cassiano Teixeira, Hospital Moinhos de Vento, Porto Alegre, Brasil; Dra.Celina Acra, Casa de Saúde Santa Lúcia, Rio de Janeiro, Brasil; Dra.Cinthia Grion, Hospital Universitário de Londrina, Londrina, Brasil; Dr. Ciro Leite Mendes, Hospital Universitário - UFPB, João Pessoa, Brasil; Dr. Cláudio Piras, Vitória Apart Hospital, Vitória, Brasil; Dr. Dalton Barros, Hospital Geral Roberto Santos, Salvador, Brasil; Dr. Ederlon Alves de Carvalho Rezende, Hospital do Servidor Público Estadual, São Paulo, Brasil; Dr. Edson Moraes, Santa Casa de Porto Alegre, Porto Alegre, Brasil; Dr. Fábio Miranda, Hospital dos Servidores do Estado, Rio de Janeiro, Brasil; Dr. Felipe Dal-Pizzol, Hospital São José, Criciúma, Brasil; Dr. Fernando Cardoso, Hospital Quinta D'or, Rio de Janeiro, Brasil; Dr. Frederico Bruzzi Carvalho, Hospital Eduardo de Menezes e Hospital Mater Dei, Belo Horizonte, Brasil; Dr. Gerson Luiz Macedo, Hospital Universitário Sul-Fluminense da Universidade Severino Sombra, Vassouras, Brasil; Dr. Gustavo G. Freitas, Hospital Barra D'Or, Rio de Janeiro, Brasil; Dr. Helio Penna Guimarães, UTI da Disciplina de Clinica Médica - UNIFESP-EPM, São Paulo, Brasil; Dr. Joel Tavares Passos, Hospital Unimed Costa do Sol, Macaé, Brasil; Dr. Jorge I. F. Salluh, Instituto Nacional de Câncer, Rio de Janeiro, Brasil; Dr. Jorge Valiatti, Hospitais Escola Padre Albino e Hospitais Escola Emilio Carlos, Ribeirão Preto, Brasil; Dr. José Jorge Soares Netto, Instituto Nacional de Câncer, Rio de Janeiro, Brasil; Dr. José Mário Meira Teles, Hospital da Bahia, Salvador, Brasil; Dr. Jose Raimundo Araujo de Azevedo, Hospital São Domingos, São Luis, Brasil; Dra. Julieta Fripp, Santa Casa de Misericórdia de Pelotas, Pelotas, Brasil; Dr. Julio Neves, Hospital da Cidade, Salvador, Brasil; Dra. Leila Rezegue, Hospital Porto Dias, Belém, Brasil; Dr. Luciano C. P. Azevedo, Hospital Sírio Libanês, São Paulo, Brasil; Dr. Luis Eduardo Fontes, Hospital Alcides Carneiro, Petrópolis, Brasil; Dr. Marcelo de Oliveira Maia, Hospital Santa Luzia, Brasilia, Brasil; Dr. Marcelo Garcia da Rocha, Santa Casa de Porto Alegre, Porto Alegre, Brasil; Dr. Marcelo Magalhães, Hospital de Clínicas de Niterói, Niterói, Brasil; Dr. Marcio Soares, Instituto Nacional de Câncer, Rio de Janeiro, Brasil; Dr. Marcos Freitas Knibel, Hospital São Lucas, Rio de Janeiro, Brasil; Dr. Mário Lúcio Alves Baptista Filho, Hospital Bandeirantes, São Paulo, Brasil; Dr. Mariza D'agostino, Hospital Nove de Julho, São Paulo, Brasil; Dr. Mauricio Velasco, Hospital Meridional, Cariacica, Brasil; Dr. Nazah C M Youssef, Hospital das Nações, Curitiba, Brasil; Dr. Otavio Gebara, Hospital Santa Paula, São Paulo, Brasil; Dr. Patricia M. Veiga de C. Mello, Hospital de Terapia Intensiva, Teresina, Brasil; Dr. Paulo Schiavetto, Santa Casa de Campo Mourão, Campo Mourão, Brasil; Dr. Pedro Caruso, Hospital A. C. Camargo, São Paulo, Brasil; Dr. Pedro Kurtz, Casa de Saúde São José, Rio de Janeiro, Brasil; Dr. Rachel Duarte Moritiz, Universidade Federal de Santa Catarina, Florianópolis, Brasil; Dr. Ricardo Pondé, Hospital Salvador, Salvador, Brasil; Dr. Rodrigo Hatum, Hospital CardioTrauma, Rio de Janeiro, Brasil; Dr. Ronaldo Gismondi, Hospital Barra D'Or, Rio de Janeiro, Brasil; Dr. Rubens Costa Filho, Hospital Pró-Cardíaco, Rio de Janeiro, Brasil; Dra. Silvia Rios Vieira, Hospital de Clinicas de Porto Alegre, Porto Alegre, Brasil; Dra. Suzana Margareth Ajeje Lobo, Hospital de Base -Fundação Faculdade Regional de Medicina de São José do Rio Preto, São José do Rio Preto, Brasil; Dra.Viviane Veiga, Unidades de Terapia Intensiva Neurológica do Hospital Beneficência Portuguesa de SP, São Paulo, Brasil.

**Chile:** Dr. Alejandro Escobar, Clínica Medicina Intensiva , Chile; Dr. Antonio Hernández, Hospital Militar de Santiago, Santiago, Chile; Dr. Carlos Ortega, Hospital Concepción, Chile; Dr. Christos Varnabas, Hospital Regional de Punta Arenas, Punta Arenas, Chile; Dr. Enrrique Camponobo, Hospital San Felipe, Chile; Dr. Francisco Arancibia Santiago, Instituto Nacional del Torax, Santiago, Chile; Dr. Gustavo Huerta, Hospital Carabineros, Santiago, Chile; Dr. Héctor Ugarte, Hospital Coquimbo, Chile; Dr. Hugo Gonzalez, Hospital San Juan, Cauquenes, Chile; Dr. Ignacio Escamilla, Hospital del Salvador, Santiago, Chile; Dr. Jorge Cantero, Hospital de Curicó, Curicó, Chile; Dr. Jorge Godoy, Hospital Sotero del Río, Cordillera, Chile; Dr. Juan Jara, Hospital Regional de Talca, Talca, Chile; Dr. Mauricio Álamo, Clínica Dávila, Santiago, Chile; Dr. Mauricio Tapia, Hospital Coyhaique, Coyhaique, Chile; Dr. Osvaldo Le Feuvre, Hospital Barros Luco, Santiago, Chile; Dr. Raúl Romero, Centro Clinico Militar Iquique, Iquique, Chile; Dr. Rodrigo Soto, Hospital Clínico FACH, Santiago, Chile; Dr. Sebastián Ugarte, Clínica Indisa, Santiago, Chile; Dr. Sergio A Pacheco, Hospital San José, Santiago, Chile; Dr. Víctor Aliste, Hospital Metropolitano de Santiago, Santiago, Chile.

**Colombia :** Dr. Abraham Ali, Fundacion Neumologica Colombiana, Bogota, Colombia; Dr. Abul Ariza, Clinica de Especialistas de La Dorada, La Dorada, Colombia; Dr. Agamenon Quintero, Instituto Médico de Alta Tecnología, Monteria, Colombia; Dr. Camilo Pizarro, Fundacion Cardiovascular de Colombia, Colombia; Dr. Carlos Cadavid, Hospital Pablo Tobon Uribe, Medellin,Colombia; Dr. Carmelo Dueñas, Clinica Universitaria San Juan de Dios e Nuevo Hospital Bocagrande, Cartagena, Colombia; Dr. Edgar Celis Bogota, Fundacion Santa Fe, Bogota, Colombia; Dr. Francisco Molina, Clinica Universitaria Bolivariana, Medellin, Colombia; Dr. Guillermo Ortiz, Hospital Santa Clara, Bogota, Colombia; Dr. Guillermo Ortiz, Hospital Santa Clara, Bogota, Colombia; Dr. Ivan Diaz, Hospital Naval, Cartagena, Colombia; Dr. Julio Cesar Duran, Clinica Valledupar, Valledupar, Colombia; Dra. Marcela Granados, Fundacion Valle del Lili, Cali, Colombia; Dr. Marco A Gonzalez, Clinica Medellin, Medellin, Colombia; Dr. Martin Carvajal, Medihelp Services, Cartagena, Colombia; Dr. Nelly Beltran, Hospital Universidad del Norte, Barranquilla, Colombia; Dr. Ruben Camargo, Clinica General del Norte, Barranquilla, Colombia; Dr. Trinidad Plata, Clinica del Country, Bogota, Colombia.

**Ecuador:** Dr. Alfredo Defilippi, Hospital Ecuador; Luis Vernaza, Guayaquil, Ecuador; Dr. Angel Zambrano, Hospital Clinica Kennedy, Guayaquil, Ecuador; Dr. Electra Moreno, Hospital de Infectología de Guayaquil, Guayaquil, Ecuador; Dr. Estuardo Salgado, Clínica La Merced, Quito, Ecuador; Dr. Freddy Morales, Hospital de SOLCA, Portoviejo, Ecuador; Dr. Gleiner Cañarte, Hospital IESS, Portoviejo, Ecuador; Dr. Gonzalo Sánchez, Hospital Luis Vernaza, Guayaquil, Ecuador; Dr. Gustavo Paredes, Hospital Enrique Garcés, Quito, Ecuador; Dr. Gustavo Pozo, Hospital General de las FFAA HG-1, Quito, Ecuador; Dr. Jean Raad, Hospital de Clínicas Pichincha, Quito, Ecuador; Dr. José Vergara, Clínica Sotomayor, Guayaquil, Ecuador; Dr. Luis Gonzalez, Hospital Luis Vernaza, Guayaquil, Ecuador; Dr. Manuel Jibaja, Hospital Eugenio Espejo, Quito, Ecuador; Dr. Marcelo Ochoa, Hospital IESS, Cuenca, Ecuador; Dr. Miguel Chung Sang, Intensiva Clínica Guayaquil, Guayaquil, Ecuador; Dr. Stenio Cevallos, Hospital Alcívar, Guayaquil, Ecuador; Dra Virginia Intriago, Hospital Verdi Cevallos, Portoviejo, Ecuador; Dra. Zolila Giler, Clínica San Gregorio, Manta, Ecuador. **Spain:** Dr. Andrés Esteban, Hospital Getafe, Madrid, Espanha; Dr. Eduardo Palencia Herrejón, Hospital "Infanta Leonor", Madrid, Espanha; Dr. Guillermo Parra, Hospital Virgen de la Veja, Murcia, Espanha; Dr. Javier Belda, Hospital Clínico Valencia, Valencia, Espanha.

**USA**: Dr. Edgar Jiménez, Orlando Regional, Orlando, U.S.A.

**Mexico:** Dr. Alberto Gutíerrez García, Beneficencia Española, S.L.P., México; Dr. Alejandro Pizaña, Angeles Santelena, México; D.F., México; Dr. Alfredo Sierra Unzueta, Hospital Español de México, México; D.F., México; Dr. Antonio Luviano, Umae 21 del IMSS, Monterrey, México; Dr. Arturo Ramírez Rosales, Hospital Muguerza, Monterrey, México; Dr. Asisclo de Jesús Villagomez Ortiz, Hospital Regional 1° Octubre, México D.F., México; Dr. Carlos Alberto Gutierrez Martinez, UMAE de Especialidades CMNO, Guadalajara Jal, México; Dr. Carlos García, Hospital Maestros Sección 50, Monterrey, México; Dr. Carlos Gonzalo Rosel Gómez, Clínica Mérida, Mérida, México; Dr. Cesar Cruz Lozano, Hospital Pemex, Tampico, México; Dr. Cesar Zuñiga, Hospital de Pemex Reynosa, Tampico, México; Dr. Daniel Arizpe Bravo, Angeles de Puebla, Puebla; México; Dr. Daniel Rodriguez, Hospital Civil de Guadalajara Juan I Menchaca, Guadalajara Jal, México; Dr. Enrique Lee Chong, Hospital IMSS Cd. Madero, Tampico, México; Dr. Enrique Mario Olivares Duran, Hospital de Alta Especialidad del Bajío, León, México; Dr. Enrique Olivares Duran, HRAE Bajío, León, México; Dr. Enrique Ramírez Gutierrez, Hospital CIMA, Sonora, México; Dr. Felipe de Jesús Pérez Rada, Hospital San José, Monterrey, México; Dr. Francisco Baranda Tovar, Instituto de Cardiología Ignacio Chávez, Mexico D.F., Mexico; Dr. Francisco Javier López Orozco, Hospital Muguerza, Saltillo, México; Dr. Guillermo Cueto Robledo, Hospital General de Mexico; Mexico D.F., Mexico; Dr. Gustavo Mendez Martinez, Angeles Villahermosa, Villahermosa, México; Dr. Ignacio Morales Camporredondo, Hospital Mocel, Mexico D.F., Mexico;

Dr. Jorge Pérez Figueroa, Hospital Milenium, Veracruz, México; Dr. José Antonio Arzate Villafaña, ISEMYM Centro Médico Toluca, Toluca, México; Dr. Jose de Jesús Bueno Almanza, Hospital de la Beneficencia, Tampico, México; Dr. José Manuel Conde Mercado, Hospital Juárez, Mexico D.F., Mexico; Dr. José Roberto Albores Medina, Beneficencia Española Torreon, Torreon, México; Dr. Juan Gerardo Esponda Prado, Hospital Angeles Pedregal, Mexico D.F., Mexico; Dr. Julio Cesar Mijangos Mendez, Hospital Civil de Guadalajara Fray Antonio Alcalde, Guadalajara, México; Dr. Juvenal Franco Granillo, Hospital ABC Observatorio e Hospital ABC Santa Fe, Mexico D.F., Mexico; Dr. Leandro B. Cervantes Ledesma, Hospital Juan Graham, Villahermosa, México; Dr. Luis Pedro Ambriz Nava, Hospital General La Raza, Mexico D.F., Mexico; Dr. Marco Antonio Cetina Camara, Hospital General Dr. Agustin O´Horan, Mérida, México; Dr. Mariano Martinez y Angel Luna, Hospital IMSS 6, Monterrey, México; Dr. Miguel Martinez Gutiérrez, Hospital de EspecialidadesUMAE CMN Siglo XXI, Mexico D.F., Mexico; Dr. Nicandro Guillen Austria, Hospital Gea González, Mexico D.F., Mexico; Dr. Oscar Palma, Hospital México CCSS, Costa Rica, México; Dr. Othon Gayosso Cruz, ISSSTE López Mateos, México; D.F., México; Dr. Pedro Gutierrez Lizardi, OCA Hospital, Monterrey, México; Dra. Raquel Mendez Reyes, ISSSTE 1° Octubre, Mexico D.F., Mexico; Dr. Raúl Carrillo, Hospital Médica Sur, Mexico D.F., Mexico; Dr. Raul Envila Fisher, Chritus Muguerza, Chihuahua; México; Dr. Raimundo Perez Cruz, Beneficencia Española, Puebla, México; Dr. Sergio Arevalo, UMAE 34 IMSS e CIMA Santa Engracia, Monterrey, México; Dr. Sergio López Estupiñan, Hospital del Carmen, Guadalajara Jal, México; Dr. Ulises Ceron Díaz, Hospital Angeles Lindavista, Mexico D.F., Mexico; Dr. Uriel Chavarria Martínez, Hospital Universitario, Monterrey, México; Dr. Victor Manuel Santana Enriquez, Hospital Metropolitano de Monterrey, Monterrey, México; Dr. Zalatiel Meycotte Luna, Hospital Angeles Interlomas, Mexico D.F., Mexico.

**Peru:**Dr. Abel Arroyo Sánchez, Hospital Victor Lazarte Echegaray, Trujillo, Peru; Dr. Jorge E. Morales Corvacho, Hospital Nacional de Policia, Lima, Peru.

**Uruguay :**Dr. Alex Pintos, Hospital Policial, Montevideo, Uruguai; Dra. Estrella Echavarría, Hospital Maciel Centro Nacional de Quemados e Hospital de Clínicas, Montevideo, Uruguai; Dr. Frank Torres, Sanatorio Cantegril, Maldonado, Uruguai; Dr. Gustavo Grecco, Sanatorio Americano, Montevideo, Uruguai; Dr. Gustavo Pittini, CAAMEPA, Pando, Uruguai; Dr. Mario Rodriguez Verde, MSP Paysandú, Paysandú, Uruguai; Dr. Nestón Campos, MSP Salto, UCIS Salto e UMIS Salto, Salto, Uruguai; Dra. Patrícia Mesa, Hospital Florida, Florida e Hospital Español, Montevideo, Uruguai; Dr. Pedro Alzugaray, CAMOC, ORAMECO, CRAMI, Uruguai; Dra. Silvia Noveri, Hospital Pasteur, Montevideo, Uruguai.
